# The DNA double-strand break repair proteins γH2AX, RAD51, BRCA1, RPA70, KU80, and XRCC4 exhibit follicle-specific expression differences in the postnatal mouse ovaries from early to older ages

**DOI:** 10.1007/s10815-024-03189-4

**Published:** 2024-07-18

**Authors:** Gunel Talibova, Yesim Bilmez, Betul Tire, Saffet Ozturk

**Affiliations:** Department of Histology and Embryology, Akdeniz University School of Medicine, Campus, 07070 Antalya, Turkey

**Keywords:** DNA double-strand break, HR repair, cNHEJ, Ovarian aging, Follicles, Oocytes, Female infertility

## Abstract

**Purpose:**

Ovarian aging is closely related to a decrease in follicular reserve and oocyte quality. The precise molecular mechanisms underlying these reductions have yet to be fully elucidated. Herein, we examine spatiotemporal distribution of key proteins responsible for DNA double-strand break (DSB) repair in ovaries from early to older ages. Functional studies have shown that the γH2AX, RAD51, BRCA1, and RPA70 proteins play indispensable roles in HR-based repair pathway, while the KU80 and XRCC4 proteins are essential for successfully operating cNHEJ pathway.

**Methods:**

Female Balb/C mice were divided into five groups as follows: Prepuberty (3 weeks old; n = 6), puberty (7 weeks old; n = 7), postpuberty (18 weeks old; n = 7), early aged (52 weeks old; n = 7), and late aged (60 weeks old; n = 7). The expression of DSB repair proteins, cellular senescence (β-GAL) and apoptosis (cCASP3) markers was evaluated in the ovaries using immunohistochemistry.

**Result:**

β-GAL and cCASP3 levels progressively increased from prepuberty to aged groups (*P* < 0.05). Notably, γH2AX levels varied in preantral and antral follicles among the groups (*P* < 0.05). In aged groups, RAD51, BRCA1, KU80, and XRCC4 levels increased (*P* < 0.05), while RPA70 levels decreased (*P* < 0.05) compared to the other groups.

**Conclusions:**

The observed alterations were primarily attributed to altered expression in oocytes and granulosa cells of the follicles and other ovarian cells. As a result, the findings indicate that these DSB repair proteins may play a role in the repair processes and even other related cellular events in ovarian cells from early to older ages.

**Supplementary information:**

The online version contains supplementary material available at 10.1007/s10815-024-03189-4.

## Introduction

In recent decades, couples are increasingly postponing parenthood to later ages due to socioeconomic reasons. However, women’s reproductive health shows age-related declines, especially in follicular reserve, oocyte quantity and quality. It is well-established that the ovarian follicular reserve is formed during prenatal and neonatal development, and that it subsequently declines. It is estimated that only approximately five hundred of one million oocytes ovulate during the reproductive lifespan of humans. The remainder are lost over time [1]. The decline in ovarian reserve is accelerated by the process of maternal aging [2, 3], resulting in complete loss in humans at an average age of 50 years [4]. The precise molecular mechanisms underlying the accelerated attrition and reduced oocyte quality observed at later reproductive ages remain to be fully elucidated. The elucidation of these mechanisms will facilitate the development of novel treatment strategies to enhance reproductive performance and even postpone the onset of menopause.

Various types of genotoxic agents derived from endogenous and exogenous factors can lead to DNA damages in eukaryotic cells. DNA double-strand breaks (DSBs) are the most dangerous among them, which can cause significant changes in genomic integrity. Although DNA damage response for DSBs is a conserved process in eukaryotic cells, there are significant distinctions between mammalian oocytes and somatic cells [5]. In more detail, oocytes show higher DNA repair proficiency and thus experience lower mutation rates when compared to somatic cells [6] because possible mutations in oocytes may be transmitted to descendants throughout generations. In somatic cells, the necessity for DNA repair in order to maintain the genome throughout the lifespan of an individual is of paramount importance. [7]. As with somatic cells, prophase-arrested oocytes [also known as germinal vesicle (GV) oocyte] are similarly susceptible to DSBs from ionizing radiation, chemotherapy, and environmental pollutants [8]. If DSBs are not repaired in an appropriate and timely manner, apoptotic cell death or cellular senescence ensues in these cells [9]. Under normal conditions, DSBs are successfully repaired by two main pathways: homologous recombination (HR) and classical non-homologous end joining (cNHEJ) [10, 11]. In the S and G2 phases of the cell cycle, the HR-based repair mechanism enables error-free repair of DSBs by utilizing the intact sister chromatid as a template [12]. In contrast, the cNHEJ pathway operates in an error-prone manner throughout cell cycle since it ligates broken DNA ends without using any template sequence [13]. In contrast to somatic cells, evidence indicates that cNHEJ is the predominant mechanism for DSB repair in GV oocytes, even during the G2 phase of the cell cycle when a sister chromatid is available [14].

The DNA damage signaling pathway mediated by ataxia telangiectasia-mutated (ATM) promotes repair of DSBs through HR-based repair mechanism. In this context, serine 139 localizing in the conserved C-terminal tail of histone H2A variant (H2AX) is phosphorylated (referred to as γH2AX after this modification) by DNA-dependent protein kinase catalytic subunit (DNA-PKcs) [15, 16]. It has been demonstrated that the number of γH2AX foci increases in primordial, primary, and secondary follicles of aged ovaries in rhesus monkeys [17]. In contrast, γH2AX intensity was found to be lower in old mouse (42–45 weeks old) GV oocytes than in young ones (5–8 weeks old), as detected in metaphase II (MII) oocytes [18]. The results demonstrate that γH2AX exhibited cell- and species-specific expression patterns.

Following the labeling of DSBs with γH2AX, the BRCA1 protein is involved in the ATM-mediated HR repair process [19]. The BRCA1 protein not only plays a role in HR repair in cooperation with RAD51, but is also involved in single-strand annealing (SSA) [20] and regulation of mitotic progression by interacting with the cell cycle checkpoint kinase 2 (CHK2) [21] and meiotic spindle assembly [22]. A deficiency of BRCA1 in mice and humans has been demonstrated to lead to ovarian aging [19] and genomic instability triggered by the p53-related cell cycle checkpoint [20]. As expected, the number of BRCA1 foci decreased in oocyte’s nucleus and granulosa cells of primordial, primary, and secondary follicles in ovaries of old rhesus monkeys [17]. In older rats [23] and buffola [24], a reduction in *BRCA1* gene expression was observed in primordial follicles compared to younger animals. Consequently, BRCA1 exhibits a decline in expression with age in follicles at various stages and their components.

Another HR repair-related component, RAD51, plays a role in the invasion of single-strand DNA (ssDNA) into duplex DNA, the pairing of homologous strands [25], and resumption of stalled replication forks [26]. In the context of increasing maternal age, GV oocytes from older bovines exhibited elevated *RAD51* mRNA levels in comparison to those from younger animals [27]. In accordance with previous findings in GV oocytes from old mice and humans [28], expression of the *Rad51* gene was found to be lower in primordial oocytes of old rats (400–450 days old) compared to immature rats (18–20 days old) [23, 29]. The findings indicate that there is an age-related discrepancy in *RAD51* gene expression among mammalian species. A reduction in mRNA levels of *RAD51* was observed in primordial follicles derived from older female rats and buffalo [23, 24]. The expression of RAD51 in primordial oocytes underscores the importance of HR-mediated DNA repair in maintaining genomic stability from early oogenesis [30].

The basic ssDNA binding protein, RPA70, is also involved in cell cycle checkpoint, DNA replication, meiotic recombination, and DSB repair [31]. Additionally, RPA protein also plays a role in nucleotide excision repair (NER), base excision repair (BER), and DNA mismatch repair (MMR) pathways [31]. In HR-based DSB repair, RPA70 covers free ssDNA ends to prevent formation of secondary structures [32]. This protein was detected in mouse oocytes at GV and MII stages [33]. Notably, RPA70 filaments are subsequently replaced by RAD51 with the help of BRCA1 and BRCA2 proteins. Nevertheless, the potential effects of ovarian aging on the distribution of RPA70 and its interaction with RAD51 in the follicles and their oocytes remain to be elucidated.

In the cNHEJ mechanism, homodimeric KU80 (also known as XRCC5) proteins first bind to each break end to recruit the remaining associated proteins, including DNA-PKcs, ligase IV (LIGIV), and the scaffold proteins such as XRCC4 [34]. Lack of *Ku80* gene in mice resulted in reduced litter size [35]. It is worth noting that KU80 is also involved in suppressing chromosomal aberrations and malignant transformation [36]. Another key cNHEJ protein, XRCC4, facilitates free end ligation by LIGIV [37]. Its absence led to embryonic lethality, probably due to the accumulation of unrepaired DSBs [38].

Beta-galactosidase (β-GAL) is a lysosomal hydrolase that cleaves terminal beta-D-galactose residues ([39], #121). β-GAL staining is a widely utilized method for the identification of senescent cells, and thus is accepted as a senescence-associated marker ([40], #122). This staining is employed to ascertain the content of senescent cells in ovarian tissue, including the stromal region and follicles, irrespective of reproductive experience [41].

Apoptosis is briefly defined as the exclusive changes in cell surface and nuclear morphological properties that result from mostly cleavage caspase 3 (cCASP3) action [42]. Given that cCASP3 plays a role in the normal execution of apoptotic cell death, it is employed as a marker of defining early stages of apoptosis. [43]. Dysregulated apoptosis in the ovaries may result in female infertility due to its impact on follicular development, oocyte quality, and hormonal regulation within the ovarian cycle [44].

The results indicate that the γH2AX, RAD51, BRCA1, and RPA70 proteins, which are involved in HR repair, and the KU80 and XRCC4 proteins, which are associated with cNHEJ, play a crucial role in the proper repair of DSBs in an age-dependent manner. As maternal age increases, the efficiency of DSB repair gradually declines, resulting in the accumulation of DSBs [45]. The molecular basis of the decline in DSB repair efficiency, and follicle and oocyte loss in later reproductive life remains unclear. In the current study, we examined spatiotemporal distribution of the β-GAL, cCASP3, γH2AX, RAD51, BRCA1, RPA70, KU80, and XRCC4 proteins in the postnatal ovaries of mice from prepuberty to older ages.

## Materials and methods

### Animals and sample collection

The paraffin sections prepared in our previous study were used in this study. [46]. Female Balb/C mice were divided into five groups as follows: Prepuberty (3 weeks old; n = 6), puberty (7 weeks old; n = 7), postpuberty (18 weeks old; n = 7), early aged (52 weeks old; n = 7), and late aged (60 weeks old; n = 7). As previously described in our studies [46–48], the groups were formed according to the following criteria: the number of each follicle type, the stromal content, and the presence of corpus luteum and corpus albicans structures. All mice were provided by the Akdeniz University Experimental Animals Application and Research Center, and they were kept under a 12-h/12-h light/dark cycle without water and food restrictions. All experimental protocols were performed in accordance with the relevant guidelines and regulations approved by the Akdeniz University Institutional Animal Care and Use Committee (Protocol no. 1037/2020.02.011). Following cervical dislocation performed immediately after ether inhalation, we dissected ovaries from mice under sterile conditions using a stereomicroscope (Zeiss, Oberkochen, Germany). Dissected ovaries were used in immunohistochemical staining after performing routine paraffin embedding process.

### Paraffin embedding

A standard procedure for processing Bouin’s-fixed paraffin-embedded tissue was used to prepare postnatal mouse ovaries [46]. After being immersed in Bouin’s solution for 12 h at + 4 °C, ovaries were dehydrated in increasing concentrations of ethanol (70%, 80%, 90%, and 100%), cleared in xylene, and then embedded in paraffin. The paraffin blocks were cut into serial sections of 5 μm thickness using a rotary microtome (Leica, Nussloch, Germany). These sections were then placed on Fisherbrand™ Superfrost™ Plus microscope slides (Thermo Scientific, Rockford, IL, USA) for immunohistochemistry staining.

### Immunohistochemical staining

The spatiotemporal distribution of β-GAL, cCASP3, γH2AX, RAD51, BRCA1, RPA70, KU80, and XRCC4 proteins was investigated in the postnatal mouse ovaries of prepubertal to late ages using immunohistochemistry, as previously described [46, 49, 50]. Sections obtained from paraffin-embedded blocks were deparaffinized in fresh xylene after being incubated in an oven at 60 °C for 1 h, and then rehydrated in a series of decreasing ethanol concentrations (from 100 to 70%). We then boiled the sections for 5 min in Tris–EDTA solution (containing 10 mM Tris base and 1 mM EDTA) for antigen retrieval in a microwave oven at 665 W. Subsequently, endogenous peroxidase activity was blocked by incubating in 3% H_2_O_2_ solution (prepared in methanol) at room temperature (RT) for 25 min. We then washed sections with phosphate buffered saline (1xPBS) and blocked them in Ultra V Block solution (Thermo Fisher Scientific, Waltham, MA, USA) for 5 min at RT to prevent possible non-specific binding. Next, the sections were incubated with γH2AX (diluted 1:1000; catalog no. 9718S, Cell Signaling Technology, Danvers, MA, USA), RAD51 (diluted 1:250; catalog no. bs-20297R, Bioss Inc., Woburn, MA, USA), BRCA1 (diluted 1:1000; catalog no. bs-0803R, Bioss Inc.), and RPA70 (diluted 1:3000; catalog no. 2267S, Cell Signaling Technology), KU80 (diluted 1:250; catalog no. MBS712948, MyBiosource, Inc., San Diego, CA, USA), XRCC4 (diluted 1: 250; catalog no. bs8510R, Bioss Inc.), cCASP3 (diluted 1:300; catalog no. 9661S, Cell Signaling Technology), β-GAL (diluted 1:1300; catalog no. 11518–1-AP, Proteintech Group, Rosemont, IL, USA) primary antibodies at + 4 °C overnight. We used isotype control IgG antibody (at the same concentration with the primary antibodies; catalog no. I5006, Sigma-Aldrich, St. Louis, MO, USA) as a negative control to determine the specificity of the primary antibodies. All primary and isotype antibodies were prepared in an antibody dilution solution (catalog no. 003118, Thermo Fisher Scientific).

After incubation with the primary antibodies, the sections were washed three times for 15 min each in 1xPBS and then incubated with biotinylated secondary antibodies (diluted 1:1000; catalog no. BA-1000, Vector Laboratories, Inc., Stuttgart, Germany) for 1 h at RT. Next, sections were treated with a streptavidin–horseradish peroxidase (HRP) complex (catalog no. TS-125-HR, Thermo Fisher Scientific) for 30 min at RT. Finally, immunoreaction was visualized with 3, 3’-diaminobenzidine (DAB) substrate (catalog no. D4168, Sigma-Aldrich) under a bright-field light microscope (Olympus, Tokyo, Japan). After washing the sections under running tap water, we counterstained them with Mayer’s hematoxylin.

Total, follicular, and cellular expression of the β-GAL, cCASP3, γH2AX, RAD51, BRCA1, RPA70, KU80, and XRCC4 proteins were evaluated using the ImageJ software program (National Institutes of Health, Bethesda, MD, USA) in the postnatal mouse ovaries from prepuberty to old ages. Microscopic images were acquired under a bright-field microscope with an Axiocam 105 color camera (Zeiss, Jena, Germany) at 200 × and 400 × original magnifications. The integrated mean values measured with the ImageJ program were divided into areas to determine the expression levels of the units. All the follicles and at least 20 cells from each structure in each section were analyzed.

### Follicular classification

The follicles were classified in accordance with the generally accepted standards [46, 51, 52]. In primordial follicles, a layer of squamous follicular cells, often referred to as pre-granulosa cells, surround GV stage oocyte. When only one layer of cuboidal follicular cells encloses GV oocyte, we defined these follicles as primary. Secondary follicles include 2–3 layers of granulosa cells with no space between granulosa cells surrounding GV oocyte. The theca layer can be observed from the antral follicle stage onwards. Preantral follicles, also known as early antral follicles, are briefly defined as having small spaces between multilayered granulosa cells and a centrally located GV oocyte. In antral follicles, a large antrum filled with follicular fluid is observed, accompanied by the presence of a GV or MII oocyte, which is located in an eccentric position. The accumulation of structurally altered granulosa and theca cells, in addition to disrupted oocyte structure, is employed to define atretic follicles. After ovulation of a mature oocyte from an antral follicle, the remaining granulosa and theca interna cells form corpus luteum after prominent intracellular changes, such as increased both lipid content and endoplasmic reticulum organelles. The corpus albicans, which consists mainly of connective tissue, is the regressed form of corpus luteum. In addition to the aforementioned structures, we have conducted immunostaining studies on the germinal epithelium surrounding the ovary, and stromal cells located mainly around follicles.

### Statistical analysis

The data were subjected to one-way analysis of variance (one-way ANOVA) across ranks, after which Tukey’s post hoc test was employed. All statistical calculations were performed using GraphPad Prism 5 software (GraphPad Software Inc., San Diego, CA, USA). Statistical significance was accepted as *P* < 0.05.

## Results

In this study, we for the first time determined cellular distribution and relative levels of the β-GAL, cCASP3, γH2AX, RAD51, BRCA1, RPA70, KU80, and XRCC4 proteins in the postnatal mouse ovaries from prepuberty to late ages**.**

### β-GAL expression

**Fig. 1:**
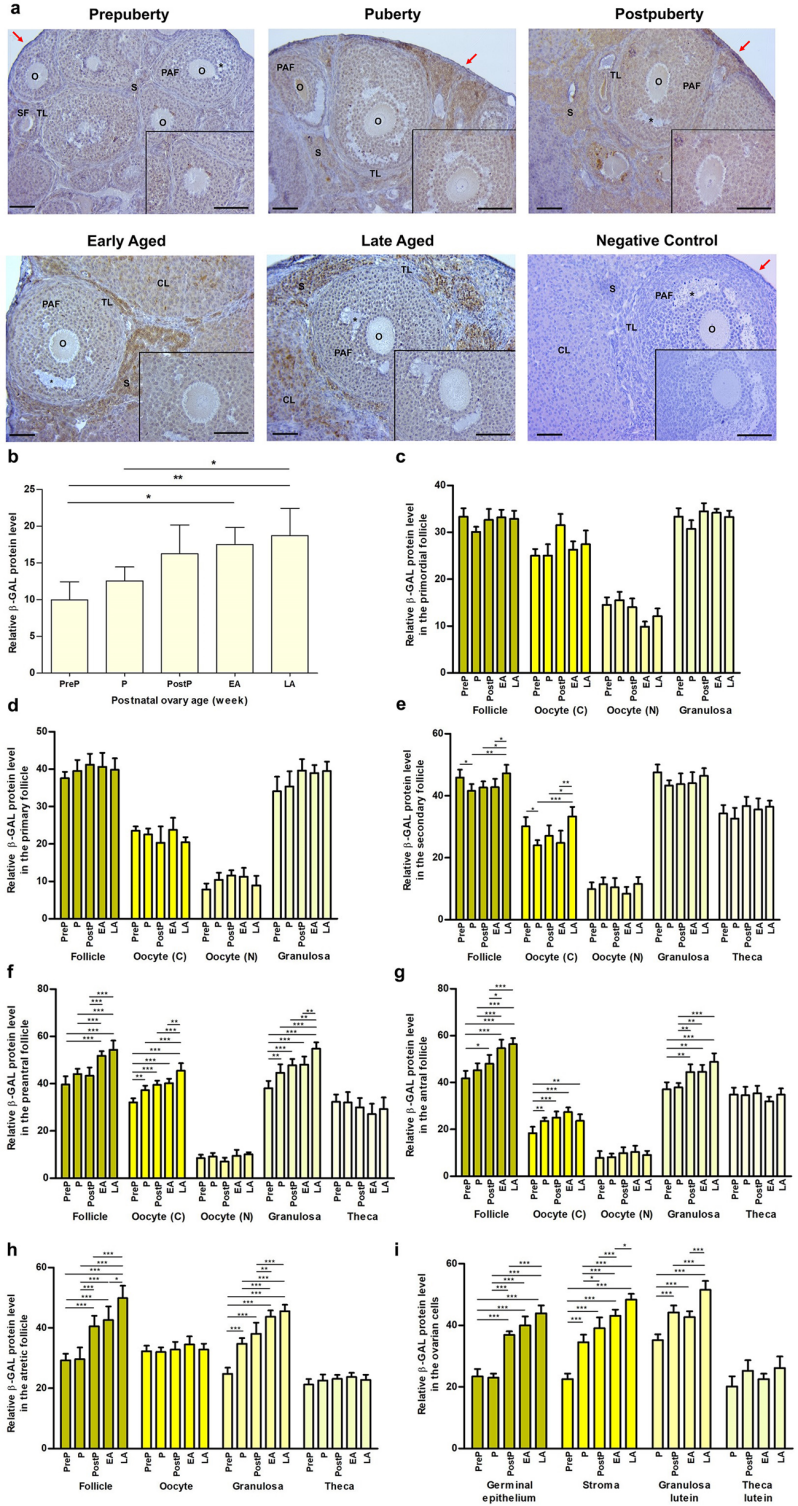
Cellular distribution and relative levels of β-GAL protein in the postnatal mouse ovaries. **a** Representative microscopic micrographs of β-GAL immunostaining of prepuberty (PreP, n = 6), puberty (P, n = 7), postpuberty (PostP, n = 7), early aged (EA, n = 7), and late aged (LA, n = 7) groups. Expression of β-GAL protein was observed in oocytes and granulosa cells of the follicles from primordial to antral stages, as well as in the other ovarian cells, including germinal epithelium, stromal cells, granulosa lutein and theca lutein cells. The asterisks indicate small spaces between granulosa cells, the red arrows show germinal epithelium. The micrographs and their inserts were captured at 200 × and 400 × original magnifications, respectively. Scale bars represent 50 µm. O, Oocyte; TL, Theca layer; S, Stroma; SF, Secondary follicle; PAF, Preantral follicle; AF Antral follicle; CL, Corpus luteum. **b** Relative β-GAL protein levels in the total area of the prepuberty, puberty, postpuberty, early aged, and late aged groups. It increased progressively from prepuberty to late aged groups (*P* < 0.05). **c-h** Relative β-GAL protein levels in **c** primordial, **d** primary, **e** secondary, **f** preantral, **g** antral, and **h** atretic follicles, and in their oocyte’s cytoplasm (C) and nucleus (N), granulosa cells, and theca cells. Although no significant change was noted in primordial and primary follicles, we observed an increasing expression in the follicles, their oocyte’s cytoplasm, and granulosa cells from prepuberty to late aged groups (*P* < 0.05). **i** Relative β-GAL protein levels in the ovarian cells located in germinal epithelium, stroma, and corpus luteum. Likewise, there was a gradual increase from the prepuberty to the late aged groups (*P* < 0.05). Data were analyzed using one-way ANOVA and Tukey’s post hoc test and are presented as the mean ± standard deviation (SD). Asterisks above the columns indicate significant differences as follows: *P < 0.05, **P < 0.01, and ***P < 0.001

The expression of β-GAL protein was observed in the nucleus and cytoplasm of oocytes and granulosa cells of the follicles at all stages, as well as in the ovarian cells, including germinal epithelium, stromal cells, granulosa lutein and theca lutein cells **(**Fig. 1a**)**. It is of note that some stromal cells in the vicinity of developing follicles exhibited a markedly intense nuclear staining in the groups from puberty to late aged. When we analyzed β-GAL expression in the total area, it progressively increased from the prepuberty to the late aged groups (*P* < 0.05; Fig. 1b). Although we observed no significant changes in primordial **(**Fig. 1c**)** and primary **(**Fig. 1d**)** follicles, β-GAL expression in secondary follicles and their oocyte’s cytoplasm reached the highest levels in the late aged group in comparison to the puberty, postpuberty, and early aged groups **(**Fig. 1e**)**. β-GAL expression in preantral **(**Fig. 1f**)** and antral **(**Fig. 1g**)** follicles, their oocytes cytoplasm, and granulosa cells increased from prepuberty to late aged groups (*P* < 0.01), with the exception of oocyte’s cytoplasm in antral follicles of late aged group. The atretic follicles and their granulosa cells **(**Fig. 1h**)**, germinal epithelium, stromal cells, and granulosa lutein cells **(**Fig. 1i**)** also demonstrated a gradual increase towards the late aged group (*P* < 0.05), except for the small decrease in granulosa lutein cells of the early aged group. These findings indicated that the majority of ovarian cells and follicles, with the exception of primordial and primary follicles, exhibited cellular senescence with advancing age.

### cCASP3 expression

**Fig. 2:**
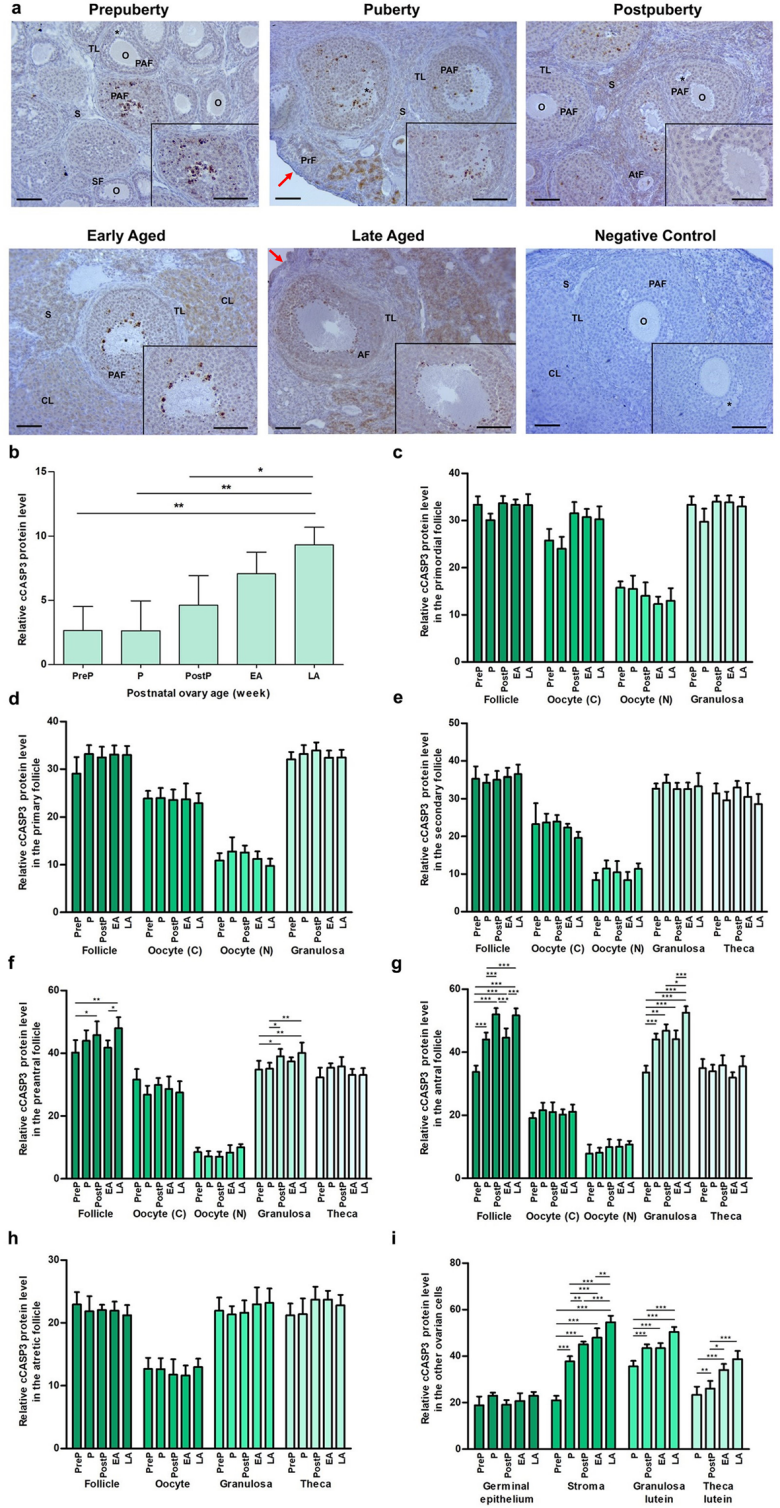
Cellular distribution and relative level of cCASP3 protein in the postnatal mouse ovaries. **a** Representative microscopic micrographs of cCASP3 immunostaining of the prepuberty (PreP, n = 6), puberty (P, n = 7), postpuberty (PostP, n = 7), early aged (EA, n = 7), and late aged (LA, n = 7) groups. cCASP3 protein was intensively expressed in granulosa cells of the growing follicles and stromal cells. The asterisks indicate small spaces between granulosa cells, the red arrows show germinal epithelium. The micrographs and their inserts were captured at 200 × and 400 × original magnifications, respectively. Scale bars represent 50 µm. O, Oocyte; TL, Theca layer; S, Stroma; SF, Secondary follicle; PAF, Preantral follicle; AF Antral follicle; AtF, Atretic follicle; CL, Corpus luteum. **b** Relative cCASP3 protein levels in the total area of the prepuberty, puberty, postpuberty, early aged, and late aged groups. It increased progressively from the prepuberty to the late aged groups (*P* < 0.05). **c-h** Relative cCASP3 protein levels in **c** primordial, **d** primary, **e** secondary, **f** preantral, **g** antral, and **h** atretic follicles, and in their oocyte’s cytoplasm (C) and nucleus (N), granulosa cells, and theca cells. Although no significant difference was observed in the primordial, primary, secondary, and atretic follicles, the cCASP3 expression reached the highest levels in the preantral and antral follicles and their granulosa cells in the late aged group compared to the early groups (*P* < 0.05). **i** Relative cCASP3 protein levels in the ovarian cells located in germinal epithelium, stroma, and corpus luteum. There was a gradual increase in the expression of stromal cells, granulosa lutein and theca lutein cells from the prepuberty to the late aged groups (*P* < 0.05). Data were analyzed using one-way ANOVA and Tukey’s post hoc test and are presented as the mean ± standard deviation (SD). Asterisks above the columns indicate significant differences as follows: *P < 0.05, **P < 0.01, and ***P < 0.001

cCASP3 was highly expressed in the granulosa cells of growing follicles, stromal cells, and granulosa lutein cells while its expression was relatively low in the remaining ovarian cells **(**Fig. 2a**)**. Upon comprehensive analysis of the overall expression in each group, it was observed that there was a discernible progression from the prepubertal to the late-aged groups (*P* < 0.05; Fig. 2b). Although no significant differences were observed between the groups in the primordial **(**Fig. 2c**)**, primary **(**Fig. 2d**)**, secondary **(**Fig. 2e**)**, and atretic follicles **(**Fig. 2h**)**, as well as in the germinal epithelium **(**Fig. 2i**)**, notable changes were identified in the remaining follicles and ovarian cells. The expression of cCASP3 in preantral and antral follicles and their granulosa cells exhibited an increasing trend from the prepuberty to the late aged groups, with the exception of a decrease observed in the early aged group (*P* < 0.05; Fig. 2f and g). Likewise, we found a progressive increase in cCASP3 levels in stromal, granulosa lutein and theca lutein cells from prepuberty to late aged groups (*P* < 0.05; Fig. 2i).

### γH2AX expression

**Fig. 3:**
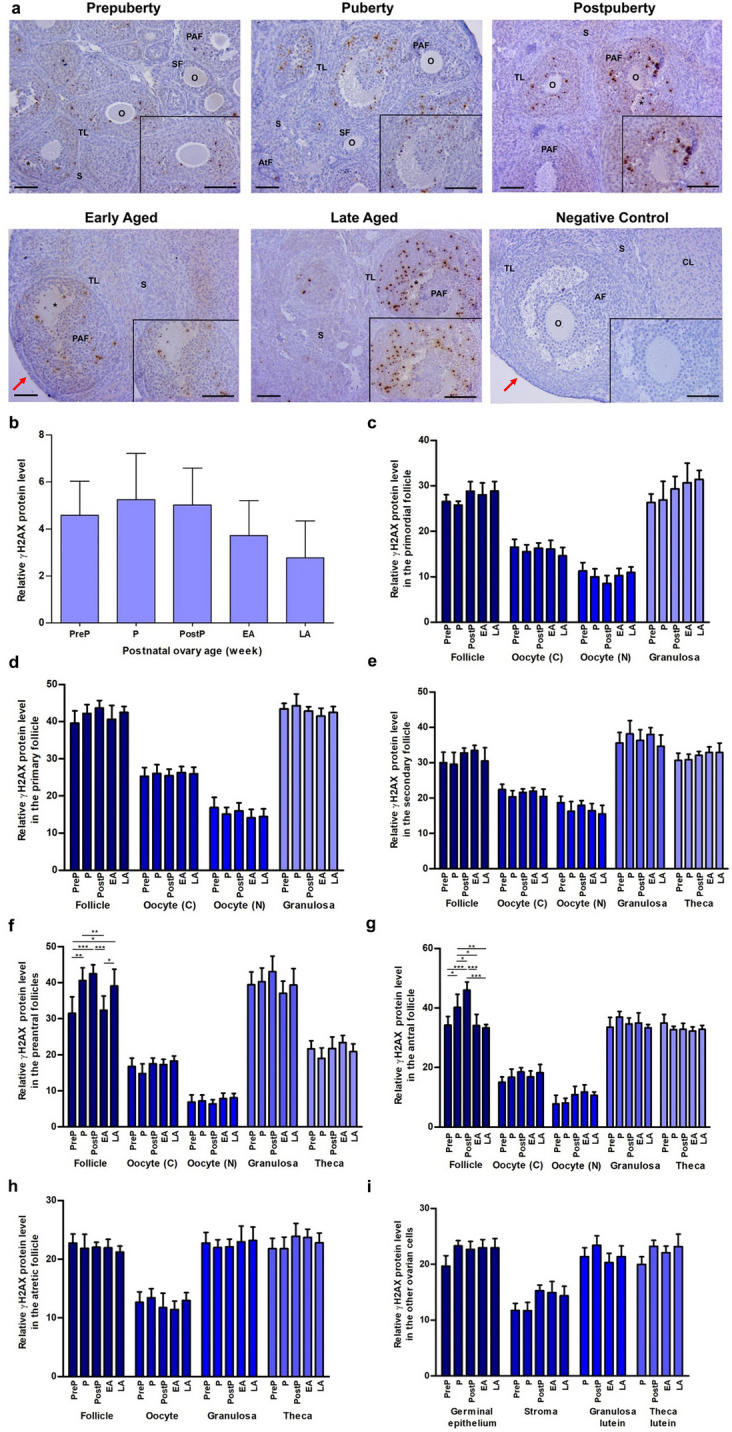
Cellular distribution and relative levels of γH2AX protein in the postnatal mouse ovaries. **a** Representative microscopic micrographs of γH2AX immunostaining of the prepuberty (PreP, n = 6), puberty (P, n = 7), postpuberty (PostP, n = 7), early aged (EA, n = 7), and late aged (LA, n = 7) groups. γH2AX protein was intensively expressed in granulosa cells of the follicles from primordial to antral stages. The asterisks indicate small spaces between granulosa cells, the red arrows show germinal epithelium. The micrographs and their inserts were captured at 200 × and 400 × original magnifications, respectively. Scale bars represent 50 µm. O, Oocyte; TL, Theca layer; S, Stroma; SF, Secondary follicle; PAF, Preantral follicle; AF, Antral follicle; AtF, Atretic follicle; CL, Corpus luteum. **b** Relative γH2AX protein levels in the total area of the prepuberty, puberty, postpuberty, early aged, and late aged groups. We observed no significant differences among the groups. **c-h** Relative γH2AX protein levels in **c** primordial, **d** primary, **e** secondary, **f** preantral, **g** antral, and **h** atretic follicles, and in their oocyte’s cytoplasm (C) and nucleus (N), granulosa cells and theca cells. Although no significant change was noted in primordial, primary, secondary, and atretic follicles, the γH2AX expression exhibited changes in preantral and antral follicles among the groups (*P* < 0.05). **i** Relative γH2AX protein levels in ovarian cells located in the germinal epithelium, stroma, and corpus luteum. We found no changes in these cells. Data were analyzed using one-way ANOVA and Tukey’s post hoc test and are presented as the mean ± standard deviation (SD). Asterisks above the columns indicate significant differences as follows: *P < 0.05, **P < 0.01, and ***P < 0.001

We observed γH2AX protein expression intensively in the nucleus of granulosa cells of the follicles from primordial to antral stages and there was low expression in the oocytes and other ovarian cells **(**Fig. 3a**)**. In analyzing relative levels of γH2AX, we found no significant differences in the total area **(**Fig. 3b**)**, primordial **(**Fig. 3c**)**, primary **(**Fig. 3d**)**, secondary **(**Fig. 3e**)**, and atretic follicles **(**Fig. 3h**)**, as well as in the other ovarian cells including germinal epithelium, stromal cells, granulosa lutein and theca lutein cells **(**Fig. 3i**)**. The expression in preantral follicles increased gradually from the prepuberty to the postpuberty (*P* < 0.01), followed by a decrease in the early aged group (*P* < 0.001), and a subsequent increase again in the late aged group (*P* < 0.05; Fig. 3f). In antral follicles, γH2AX expression increased from the prepuberty to the postpuberty (*P* < 0.05), and then decreased toward the late aged group (*P* < 0.001; Fig. 3g).

### RAD51 expression

**Fig. 4:**
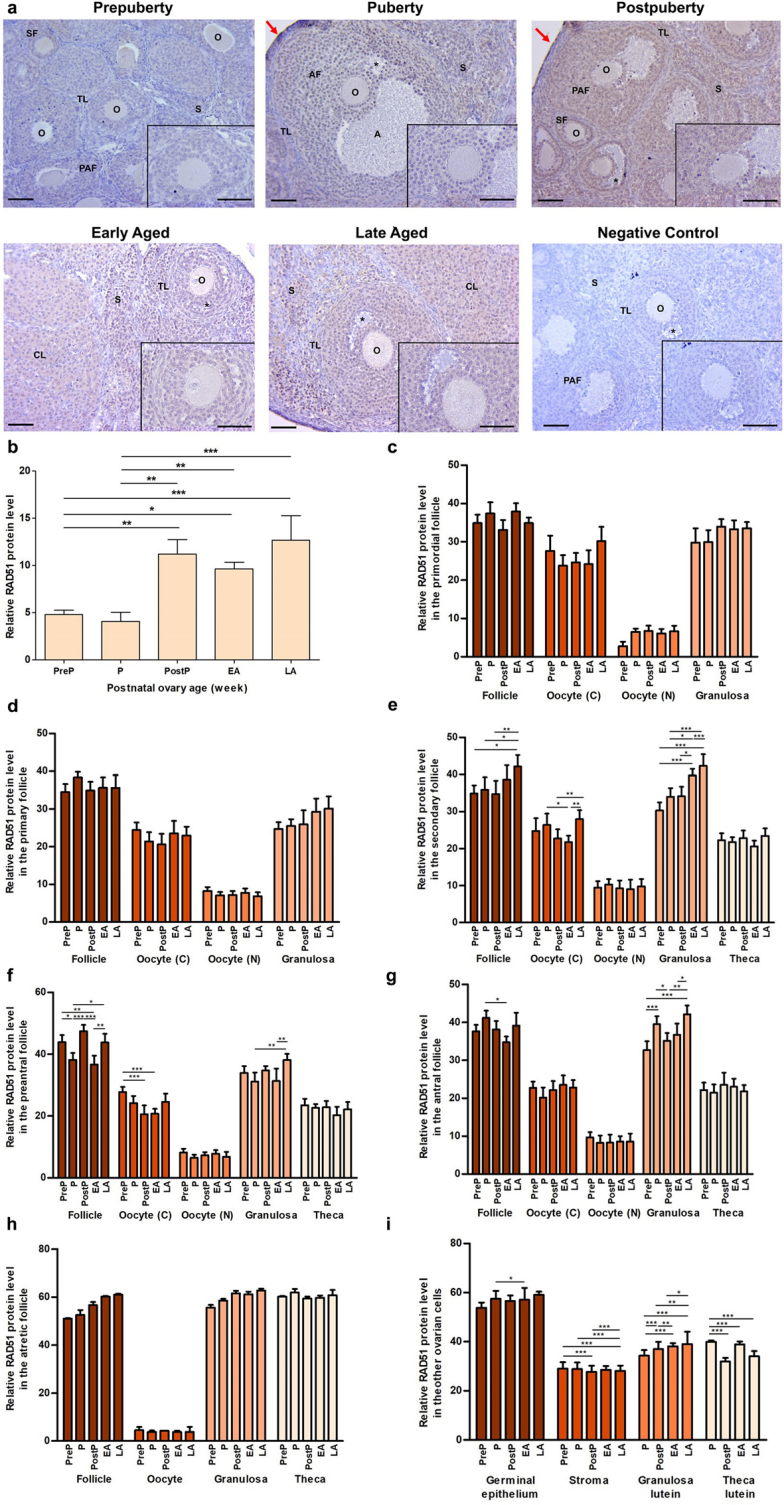
Cellular distributions and relative levels of RAD51 protein in the postnatal mouse ovaries. **a** Representative microscopic micrographs of RAD51 immunostaining of prepuberty (PreP, n = 6), puberty (P, n = 7), postpuberty (PostP, n = 7), early aged (EA, n = 7), and late aged (LA, n = 7) groups. The RAD51 protein was intensively expressed in granulosa cells of the follicles from primordial to antral stages, and in stromal cells. The asterisks indicate small spaces between granulosa cells, the red arrows show the germinal epithelium. The micrographs and their inserts were captured at 200 × and 400 × original magnifications, respectively. The scale bars represent 50 µm. O, Oocyte; TL, Theca layer; S, Stroma; SF, Secondary follicle; PAF, Preantral follicle; AF, Antral follicle; CL, Corpus luteum. **b** Relative RAD51 protein levels in the total area of the prepuberty, puberty, postpuberty, early aged, and late aged groups. It was at high levels in the postpuberty, early aged, and late aged groups (*P* < 0.05). **c-h** Relative RAD51 protein levels in **c** primordial, **d** primary, **e** secondary, **f** preantral, **g** antral, and **h** atretic follicles, and in cytoplasm (C) and nucleus (N) of oocytes, granulosa cells, and theca cells. Although no significant change was observed in primordial, primary, and atretic follicles, RAD51 expression in secondary, preantral, and antral follicles exhibited variations between the groups (*P* < 0.05). **i** Relative RAD51 protein levels in ovarian cells located in the germinal epithelium, stroma, and corpus luteum. Although there was a gradual increase in granulosa lutein cells from the prepuberty to the late aged groups (*P* < 0.05), we observed expressional changes in the remained cells (*P* < 0.05). Data were analyzed using one-way ANOVA and Tukey’s post hoc test and are presented as the mean ± standard deviation (SD). Asterisks above the columns indicate significant differences as follows: *P < 0.05, **P < 0.01, and ***P < 0.001

We observed strong RAD51 expression in the cytoplasm and nucleus of granulosa cells and stromal cells, as well as there was weak expression in oocytes of the follicles and other ovarian cells **(**Fig. 4a**)**. Total RAD51 expression was at low levels in the prepuberty and the puberty groups (*P* < 0.05), and increased in the postpuberty, early aged, and late aged groups (*P* < 0.01; Fig. 4b). Although we found no differences between the groups in primordial **(**Fig. 4c**)**, primary **(**Fig. 4d**),** and atretic follicles **(**Fig. 4h**)**, there were significant changes in secondary, preantral and antral follicles as well as in the ovarian cells. The secondary follicles, their oocyte’s cytoplasm and granulosa cells expressed RAD51 at the highest level in the late aged group in comparison to the early groups (*P* < 0.05; Fig. 4e). We also determined expressional changes in preantral **(**Fig. 4f**)** and antral follicles **(**Fig. 4g**)** and their granulosa cells between the groups (*P* < 0.05). Likewise, expression variations were observed in germinal epithelium (*P* < 0.05), stromal (*P* < 0.001) and theca lutein cells (*P* < 0.001). A gradual increase was observed in granulosa lutein cells from the prepubertal to the late aged groups (*P* < 0.05; Fig. 4i).

### BRCA1 expression

**Fig. 5:**
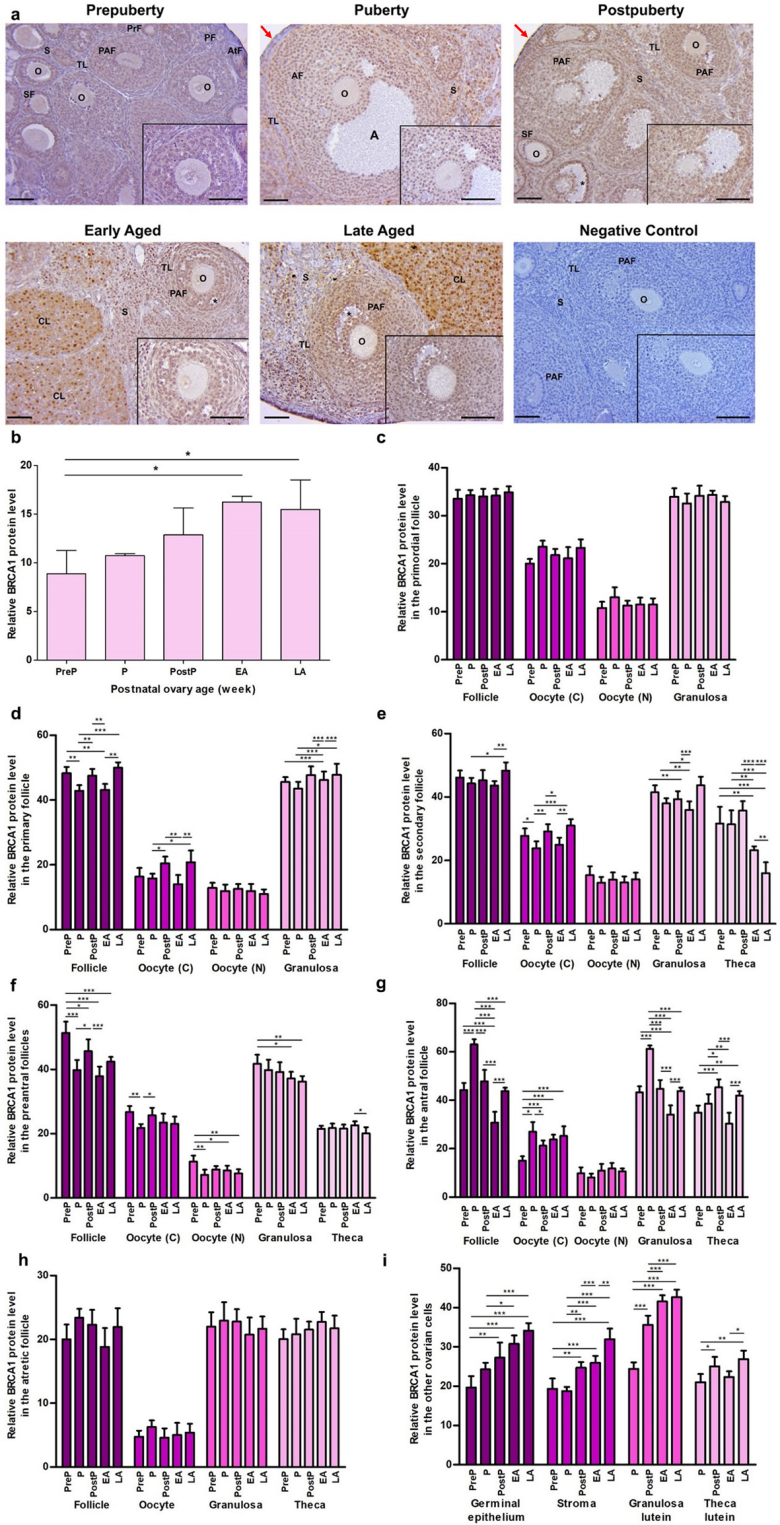
Cellular distribution and relative levels of BRCA1 protein in the postnatal mouse ovaries. **a** Representative microscopic micrographs of BRCA1 immunostaining of prepuberty (PreP, n = 6), puberty (P, n = 7), postpuberty (PostP, n = 7), early aged (EA, n = 7), and late aged (LA, n = 7) groups. BRCA1 protein was intensely expressed in granulosa cells of the growing follicles, stromal cells, and granulosa lutein cells. The asterisks indicate small spaces between granulosa cells, the red arrows show germinal epithelium. The micrographs and their inserts were captured at 200 × and 400 × original magnifications, respectively. The scale bars represent 50 µm. O, Oocyte; TL, Theca layer; S, Stroma; PrF, Primary follicle; SF, Secondary follicle; PAF, Preantral follicle; AF, Antral follicle; AtF, Atretic follicle; CL, Corpus luteum. **b** Relative BRCA1 protein levels in the total area of the prepuberty, puberty, postpuberty, early aged, and late aged groups. It gradually increased from the prepuberty to the early/late aged groups (*P* < 0.05). **c-h** Relative BRCA1 protein levels in **c** primordial, **d** primary, **e** secondary, **f** preantral, **g** antral, and **h** atretic follicles, and in their oocyte’s cytoplasm(C) and nucleus (N), granulosa cells and theca cells. Although no significant change was noted in primordial and atretic follicles, BRCA1 expression decreased in primary, secondary, preantral, and antral follicles from the prepuberty to the early/late aged groups (*P* < 0.05). **i** Relative BRCA1 protein levels in ovarian cells located in the germinal epithelium, stroma, and corpus luteum. We observed that there was a gradual increase in germinal epithelium from the prepuberty to the late aged groups (*P* < 0.05), and the stromal cells, granulosa lutein and theca lutein cells had highest levels in the late aged group (*P* < 0.05). Data were analyzed using one-way ANOVA and Tukey’s post hoc test and are presented as the mean ± standard deviation (SD). Asterisks above the columns indicate significant differences as follows: *P < 0.05, **P < 0.01, and ***P < 0.001

BRCA1 immunostaining was observed to be intensely expressed in the nuclei of granulosa cells of growing follicles, granulosa lutein cells, and stromal cells **(**Fig. 5a**)**. However, there was weak cytoplasmic and nuclear expression in oocytes of the follicles and other ovarian cells. The relative BRCA1 protein level gradually increased from the prepuberty to the early/late aged groups (*P* < 0.05; Fig. 5b). Despite minor variations in expression, no discernible alterations were observed in primordial (Fig. 5c) and atretic follicles (Fig. 5h). The expression of BRCA1 was found to vary between the groups in primary follicles, cytoplasm of their oocytes and granulosa cells (*P* < 0.05; Fig. 5d). We found low expression of BRCA1 in secondary (Fig. 5e**)** and preantral follicles (Fig. 5f**)**, cytoplasm and nucleus of their oocytes, as well as in theca cells of the early or the late aged group compared to the early groups (*P* < 0.05), with the exception of cytoplasm and nucleus of oocytes of the puberty group. The antral follicles and their granulosa and theca cells except for oocyte’s cytoplasm had the lowest expression in the early or late aged group when compared to the remaining groups (*P* < 0.05; Fig. 5g). The BRCA1 expression in oocyte’s cytoplasm of antral follicles increased gradually from the prepuberty to the late aged groups (*P* < 0.05), except for the puberty group that possessed the highest expression (*P* < 0.05). While the germinal epithelium (*P* < 0.05) and granulosa lutein cells (*P* < 0.001) exhibited progressively increased BRCA1 expression from the prepuberty to the late aged groups, the stromal cells (*P* < 0.01) and theca lutein cells (*P* < 0.05) had the highest expression in the late aged group, and a distinction was observed between the other groups **(**Fig. 5i**)**.

### RPA70 expression

**Fig. 6:**
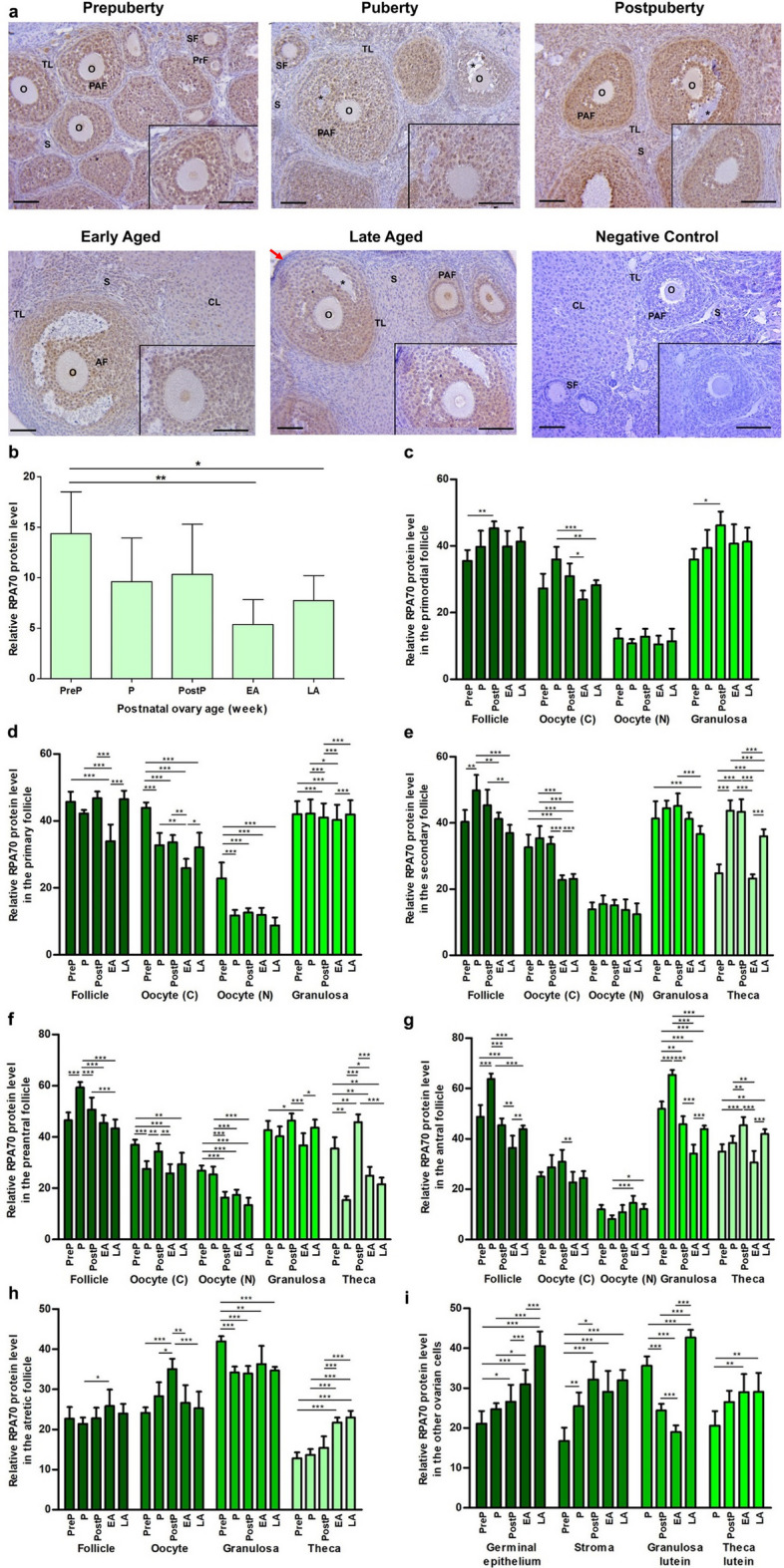
Cellular distribution and relative levels of RPA70 protein in the postnatal mouse ovaries. **a** Representative microscopic micrographs of RPA70 immunostaining of prepuberty (PreP, n = 6), puberty (P, n = 7), postpuberty (PostP, n = 7), early aged (EA, n = 7), and late aged (LA, n = 7) groups. RPA70 protein was intensely expressed in granulosa cells and nucleus of oocytes of the follicles at different developmental stages as well as in most stromal cells. The asterisks indicate small spaces between granulosa cells, the red arrows show the germinal epithelium. The micrographs and their inserts were captured at 200 × and 400 × original magnifications, respectively. The scale bars represent 50 µm. O, Oocyte; TL, Theca layer; S, Stroma; PrF, Primary follicle; SF, Secondary follicle; PAF, Preantral follicle; AF, Antral follicle; AtF, Atretic follicle; CL, Corpus luteum. **b** Relative RPA70 protein levels in the total area of the prepuberty, puberty, postpuberty, early aged, and late aged groups. It decreased in the early/late aged groups compared to the early groups (*P* < 0.05). **c-h** Relative RPA70 protein levels in **c** primordial, **d** primary, **e** secondary, **f** preantral, **g** antral, and **h** atretic follicles, and in oocyte’ cytoplasm (C) and nucleus (N), granulosa cells, and theca cells. We observed significant changes in RPA70 expression of these follicles and their components (*P* < 0.05). **i** Relative RPA70 protein levels in ovarian cells located in the germinal epithelium, stroma, and corpus luteum. The RPA70 expression significantly increased in germinal epithelium, granulosa lutein and theca lutein cells in the early/late aged group when compared to the other groups (*P* < 0.05). Data were analyzed using one-way ANOVA and Tukey’s post hoc test and are presented as the mean ± standard deviation (SD). Asterisks above the columns indicate significant differences as follows: *P < 0.05, **P < 0.01, and ***P < 0.001

In the postnatal mouse ovaries from the prepuberty to the late aged groups, we observed an intense nuclear and a moderate cytoplasmic RPA70 expression in oocytes and granulosa cells of the follicles form primordial to antral stages, as well as in most stromal cells (Fig. 6a). RPA70 expression decreased in the remained ovarian cells, including some stromal cells, theca cells, germinal epithelium, granulosa lutein and theca lutein cells. When we analyzed total RPA70 expression in the groups, we found a decreased expression in the early/late aged groups when compared to the early groups (*P* < 0.05; Fig. 6b). The RPA70 expression in primordial follicles and their oocyte’s cytoplasm and granulosa cells increased from the prepuberty to the postpuberty, and then decreased in the early or the late aged group (*P* < 0.05; Fig. 6c). The primary and secondary follicles, their oocytes, granulosa and theca cells exhibited a decreased expression in the early or the late aged group compared to the early groups (*P* < 0.05; Fig. 6d and e). In the preantral and antral follicles, their oocytes, granulosa cells, and theca cells, we observed significant decreases in the early or the late aged group in comparison to the early groups, with the exception of theca cells of preantral follicles in the puberty group as well as in nucleus of oocytes of antral follicles (*P* < 0.05; Fig. 6f and g). Although we found a higher expression in the early and the late aged groups than in the remaining groups and a gradual increase of RPA70 expression in theca cells from the prepuberty to the late aged groups (*P* < 0.001), there were expressional changes in oocytes and granulosa cells of atretic follicles (*P* < 0.05; Fig. 6h). The germinal epithelium and theca lutein cells exhibited an increasing trend in RPA70 expression from the prepuberty to the early/late aged groups (*P* < 0.05; Fig. 6i). The stromal cells (*P* < 0.05) and granulosa lutein cells (*P* < 0.001) exhibited the highest expression in the postpuberty and the late aged groups, respectively, when compared to the other groups **(**Fig. 6i).

### KU80 expression

**Fig. 7:**
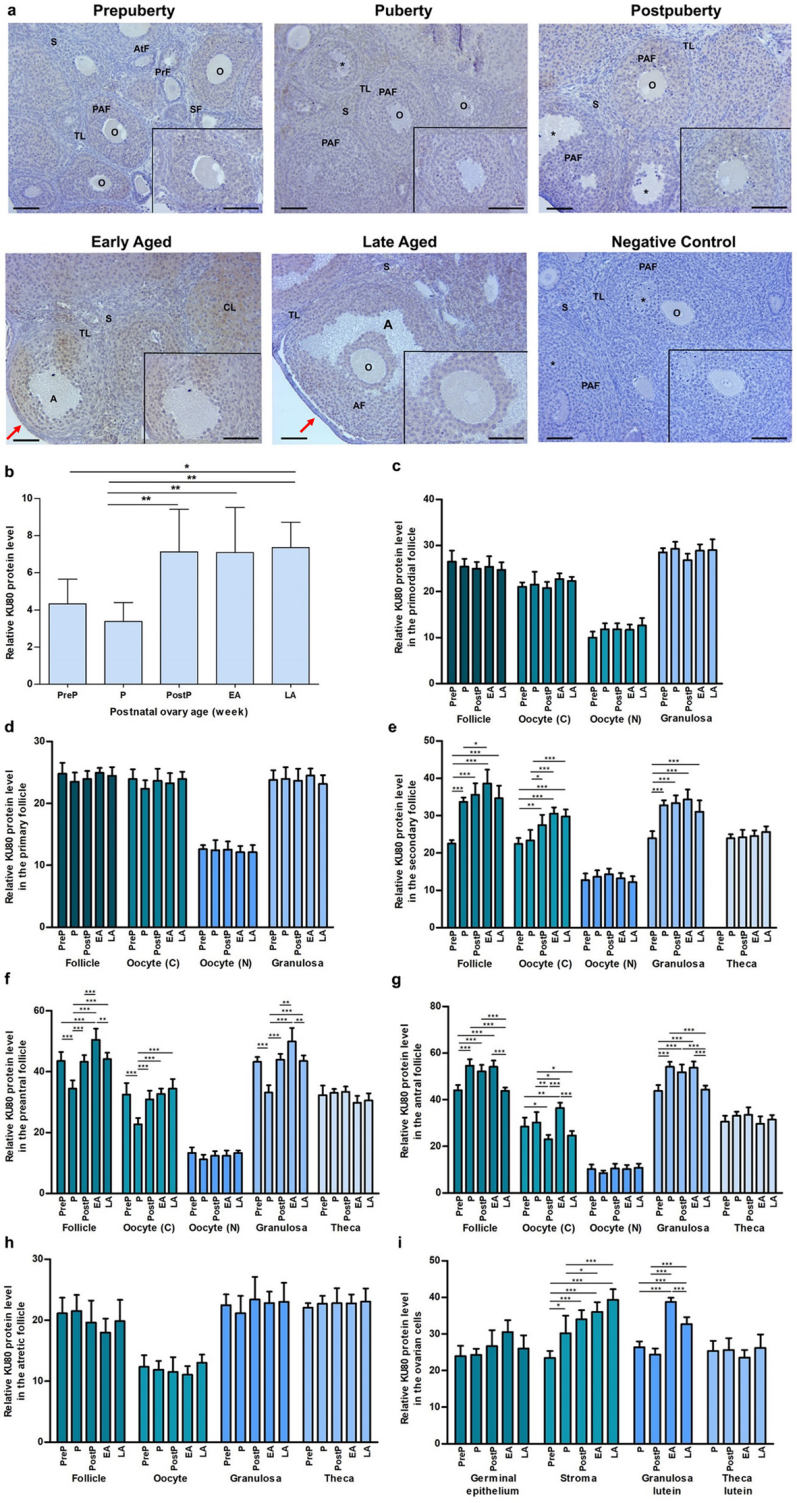
Cellular distribution and relative levels of KU80 protein in the postnatal mouse ovaries. **a** Representative microscopic micrographs of KU80 immunostaining of prepuberty (PreP, n = 6), puberty (P, n = 7), postpuberty (PostP, n = 7), early aged (EA, n = 7), and late aged (LA, n = 7) groups. The KU80 protein was expressed in granulosa cells of the follicles at the different developmental stages as well as in granulosa lutein and theca lutein cells. The asterisks indicate small spaces between granulosa cells, the red arrows show germinal epithelium. The micrographs and their inserts were captured at 200 × and 400 × original magnifications, respectively. The scale bars represent 50 µm. O, Oocyte; TL, Theca layer; S, Stroma; PrF, Primary follicle; SF, Secondary follicle; PAF, Preantral follicle; AtF, Atretic follicle; CL, Corpus luteum. **b** Relative KU80 protein levels in the total area reached high levels in the postpuberty, early aged, and late aged groups (*P* < 0.05). **c-h** The relative KU80 protein levels in **c** primordial, **d** primary, **e** secondary, **f** preantral, **g** antral, and **h** atretic follicles, and in oocyte’s cytoplasm (C) and nucleus (N), granulosa cells and theca cells. We observed an increasing trend of KU80 expression in secondary, preantral and antral follicles and their components in the early or late aged group (*P* < 0.05). **i** The relative KU80 protein levels in ovarian cells located in germinal epithelium, stroma, and corpus luteum. The KU80 expression in stromal cells and granulosa lutein cells significantly increased in the early or late aged group compared to the other groups (*P* < 0.05). Data were analyzed by one-way ANOVA and Tukey’s post hoc test and are presented as the mean ± standard deviation (SD). Asterisks above the columns indicate significant differences as follows: *P < 0.05, **P < 0.01, and ***P < 0.001

The expression of KU80 was observed in the cytoplasm of granulosa cells of follicles at different stages, as well as in granulosa lutein and theca lutein cells of corpora lutea. Additionally, weak expression was noted in other ovarian cells, including oocytes of follicles and stromal cells (Fig. 7a). When analyzing total expression of KU80, we found significantly higher expression in the postpuberty, early aged, and late aged groups than in the prepuberty and puberty groups (*P* < 0.05; Fig. 7b). No significant alterations were found in primordial (Fig. 7c), primary (Fig. 7d), atretic follicles (Fig. 7h), and their respective components. KU80 expression in secondary follicles, their oocyte’s cytoplasm and granulosa cells increased gradually from the prepuberty to the early or late aged groups (*P* < 0.05; Fig. 7e). In the preantral follicles, their oocyte’s cytoplasm and granulosa cells, KU80 expression decreased from the prepuberty to the puberty groups (*P* < 0.001) and increased toward the early or the late aged group (*P* < 0.01; Fig. 7f). However, we observed a changing expression of KU80 protein in antral follicles, their oocyte’s cytoplasm and granulosa cells between the groups (*P* < 0.05; Fig. 7g). The KU80 levels in stromal cells and granulosa lutein cells increased from the prepuberty to the early/late aged groups, with the exception of minor decrease in granulosa lutein cells of the postpuberty group (*P* < 0.05; Fig. 7i).

### XRCC4 expression

**Fig. 8:**
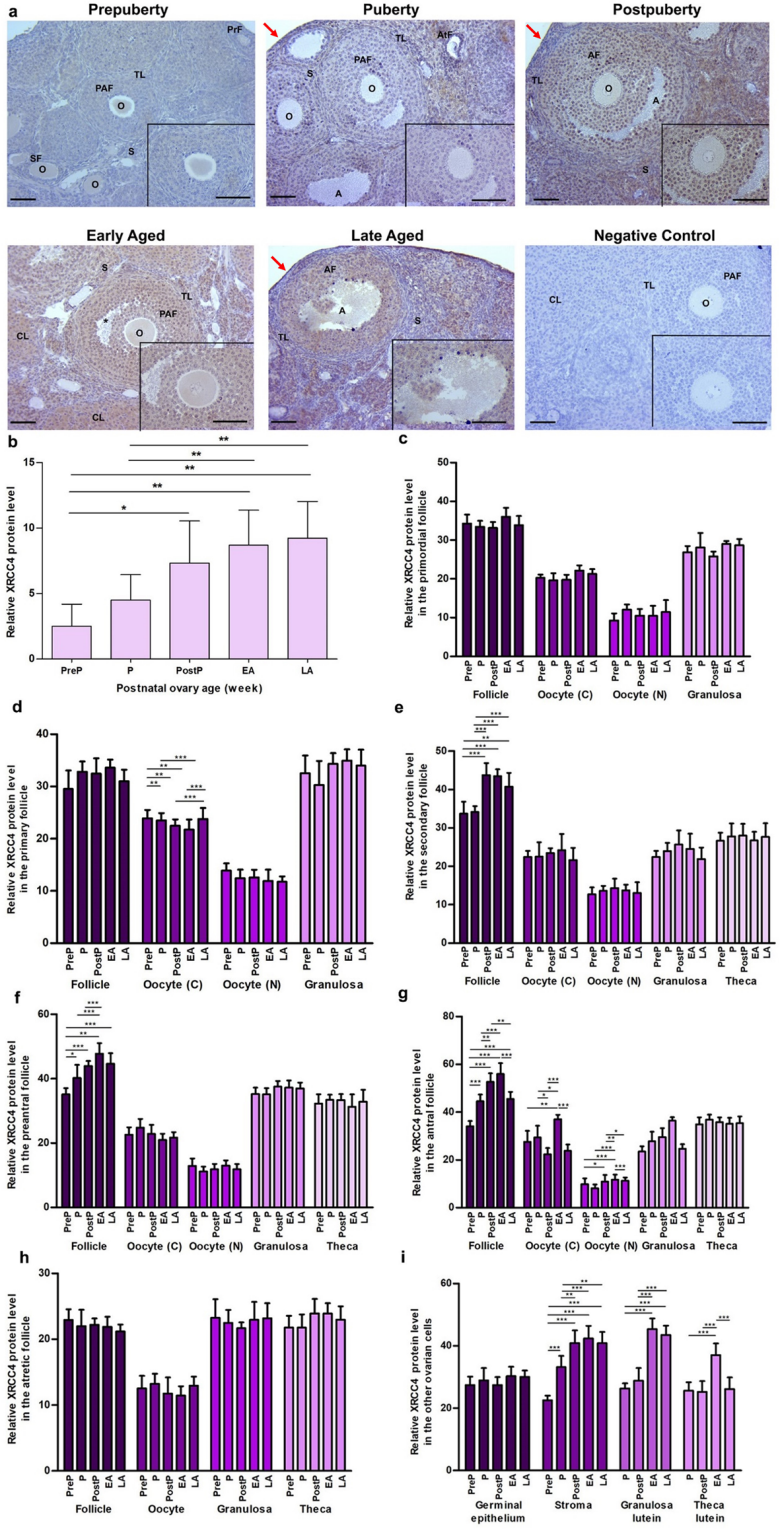
Cellular distribution and relative levels of XRCC4 protein in the postnatal mouse ovaries. **a** Representative microscopic micrographs of XRCC4 immunostaining of prepuberty (PreP, n = 6), puberty (P, n = 7), postpuberty (PostP, n = 7), early aged (EA, n = 7), and late aged (LA, n = 7) groups. XRCC4 protein was intensely expressed in oocytes and granulosa cells of the follicles at different developmental stages as well as in granulosa lutein and theca lutein cells. The asterisks indicate small spaces between granulosa cells, the red arrows show germinal epithelium. The micrographs and their inserts were captured at 200 × and 400 × original magnifications, respectively. Scale bars represent 50 µm. O, Oocyte; TL, Theca layer; S, Stroma; SF, Secondary follicle; PAF, Preantral follicle; AF, Antral follicle; AtF, Atretic follicle; CL, Corpus luteum. **b** The relative XRCC4 protein levels in the total area. It gradually increased from the prepuberty to the late aged groups (*P* < 0.05). **c-h** The relative XRCC4 protein levels in **c** primordial, **d** primary, **e** secondary, **f** preantral, **g** antral, and **h** atretic follicles, and in oocyte’s cytoplasm (C) and nucleus (N), granulosa cells, and theca cells. Although we found no changes in primordial and atretic follicles, there was an increasing trend of XRCC4 expression in primary, secondary, preantral, and antral follicles toward to the early or late aged group when compared to the early groups (*P* < 0.05). **i** The relative XRCC4 protein levels in ovarian cells located in the germinal epithelium, stroma, and corpus luteum. It increased in stromal cells, granulosa lutein and theca lutein cells from the prepuberty to the late aged groups (*P* < 0.01). Data were analyzed using one-way ANOVA and Tukey’s post hoc test and are presented as the mean ± standard deviation (SD). Asterisks above the columns indicate significant differences as follows: *P < 0.05, **P < 0.01, and ***P < 0.001

We found strong XRCC4 expression in cytoplasm and nucleus of granulosa cells and oocytes of the follicles from primordial to antral stages, especially in the postpuberty, early aged, and late aged groups (Fig. 8a). When analyzing total expression in the postnatal ovaries, a gradual increase in the expression was observed from the prepuberty to the late aged groups (*P* < 0.05; Fig. 8b). Although no significant changes were noted in primordial (Fig. 8c) and atretic follicles (Fig. 8h), there were significant changes in the remaining follicles and other ovarian cells. XRCC4 expression in oocyte’s cytoplasm of primary follicles gradually decreased from the prepuberty to the early aged groups (*P* < 0.001), and then increased in the late aged group (*P* < 0.01; Fig. 8d). Secondary follicles of the postpuberty, early and late aged groups had higher XRCC4 expression than in the prepuberty and puberty groups (*P* < 0.01; Fig. 8e). The preantral (*P* < 0.05; Fig. 8f) and antral follicles (*P* < 0.01; Fig. 8g) exhibited an increasing expression trend from the prepuberty to the early aged groups, and then it decreased in the late aged group. We also found changing XRCC4 expression in oocyte’s cytoplasm and nucleus of antral follicles (*P* < 0.05; Fig. 8g). In the stromal, granulosa lutein and theca lutein cells, we found a significantly increased expression of XRCC4 from the prepuberty to the early or late aged groups (*P* < 0.01), except for a minor decrease in theca lutein cells of the postpuberty group **(**Fig. 8i**)**.

## Discussion

The current study revealed a significant increase in the lysosomal enzyme senescence-associated β-GAL and the effector protease cCASP3 in mouse ovaries from early to old ages. The progressive increase in β-GAL and cCASP3 levels with age was largely due to their upregulated expression in growing follicles and other ovarian cells. Furthermore, the HR components γH2AX, RAD51, BRCA1, and RPA70 proteins (Supplementary Figs. 1 and 2) and the cNHEJ components KU80 and XRCC4 (Supplementary Figs. 1 and 3) exhibited the most significant changes in the postnatal ovaries from early to old ages. The primary rationale for this phenomenon can be attributed to the altered numbers of follicles at different stages of development and the luteal structures, such as corpus luteum and corpus albicans, in aging ovaries, as previously demonstrated in our research [47].

β-GAL expression is a commonly employed biomarker for the determination of cellular senescence [53, 54]. In accordance with the selected age range of the mice in our study, β-GAL expression demonstrated a progressive increase in the early (52 weeks old) and late aged (60 weeks old) groups, relative to the earlier ages. The observed increases were largely attributable to enhanced expression in the oocytes and granulosa cells of the secondary, preantral, antral and atretic follicles as well as in the germinal epithelium, stromal and granulosa lutein cells. In accordance with our findings, a previous study observed an increase in β-GAL activity in aged mouse ovaries [55]. It is noteworthy that the accumulation of lipofuscin and β-GAL foci was observed exclusively in stromal cells. Similarly, a recent study by Maruyama et al., (2023) observed the accumulation of senescent cells in the stroma of aged ovaries in mice (8–10 months old) in addition to some follicles, likely atretic follicles [49]. The observed discrepancies between these studies may be attributed to differences in the age and strains of the mice and the disparate methodologies employed in defining senescent cells.

Cellular aging can result in cell cycle arrest or apoptosis, as evidenced by the progressive increase in cCASP3 expression observed during ovarian aging [56]. In the present study, this was particularly evident in granulosa cells of preantral and antral follicles, as well as in stromal cells, granulosa lutein and theca lutein cells. Despite the absence of differences between the postnatal ovary groups in theca lutein cells of the corpus luteum, the increased apoptosis in these cells may be attributed to senescence-independent mechanisms, such as the regression of this structure to the corpus albicans by the process of luteolysis [57]*.* The increased apoptosis observed in granulosa cells of late-stage follicles, stromal cells, and luteal cells may result from elevated ROS levels during physiological aging [58]. This phenomenon has been shown to induce mitochondria-linked apoptosis [58, 59].

The prolonged arrest of mammalian oocytes at the diplotene stage of first meiotic prophase, which can persist for months in mice and decades in humans, is closely associated with the accumulation of a considerable amount of DNA damage [60]. In the processes of DNA replication and transcription, double-strand DNA (dsDNA) is unwound to serve as a template for DNA and RNA syntheses, respectively. This process facilitates the exposure of ssDNA regions to damaging factors, which may result in the deposition of DNA lesions [61]. γH2AX is employed as a biomarker to identify DSBs resulting from exposure to cytotoxic chemical agents, environmental factors, and physical stressors [62]. Following phosphorylation at serine 139 by the (ATM) or ATM-Rad3-related (ATR) kinase during the phosphoinositide 3-kinase (PI3K) pathway, γH2AX plays a pivotal role in the recruitment of the DSB repair proteins to damaged sites [17, 63]. Zhang and colleagues observed an increase in the number of γH2AX foci in primordial, primary, and secondary follicles increased from young (3–4 years old) to old (18–19 years old) monkeys [17]. In addition, the number of γH2AX foci in granulosa cells of secondary follicles and antral follicles increased in middle-aged (7–8 years old) monkeys compared to the young ones. Herein, we found a significant decrease in the γH2AX expression only in preantral and antral follicles of the aged groups in comparison to the early groups. The observed discrepancy may be attributed to the utilization of disparate methodologies for the staining of γH2AX protein, the age of the animals, and/or species-specific variations. In the light of the findings from these studies, the elevated γH2AX levels/foci observed in the follicles from primordial to antral stages in aged ovaries may be indicative of accelerated follicular loss (or follicular atresia) in the later stages of the ovary’s lifespan [64].

Apoptosis is a process that eliminates non-functional cells in the ovaries at various stages of development, from early to late ages. This mechanism is also essential in follicular atresia, which involves both oocytes and granulosa cells [65, 66]. Dysregulation of apoptosis is a contributing factor in the development of female infertility, as it negatively impacts follicular development and oocyte numbers [44]. Furthermore, an increase in apoptosis in granulosa cells has a negative impact on both the pregnancy and live birth rates in the context of in vitro fertilization (IVF) [67].

It is established that the enzyme cCASP3 is capable of initiating the activation of endogenous endonucleases, which are responsible for the fragmentation of DNA [68]. Accordingly, cCASP3 was found to be highly expressed in granulosa cells of atretic follicles in pigs [69], as well as in the ovary of buffalo [70]. A significant increase in the rate of apoptosis was observed in mural granulosa cells of women with diminished ovarian reserve, which resulted in a reduction in the number of oocytes and embryos produced in IVF centers [71]. In other words, uncontrolled apoptosis of granulosa cells has a detrimental effect on both the probability of a successful pregnancy and the likelihood of a live birth [67]. In the present study, we observed an increased expression of cCASP3 in preantral and antral follicles, as well as in stromal cells, granulosa lutein and theca lutein cells, in old ages. This finding indicates that the elimination of granulosa cells and luteal cells in the corpus luteum is predominantly achieved through the apoptotic pathway during the aging process, with some stromal cells also undergoing this process. A reduction in reproductive hormones, such as estrogen and progesterone, during ovarian aging [72] may contribute to the increased apoptosis observed in ovarian cells, including estrogen and/or progesterone receptors, due to ability of these hormones to repress the apoptotic process [73–76]. It is also possible to remove other ovarian cells, including oocytes and granulosa cells, through alternative mechanisms, such as autophagy [77].

The RAD51, BRCA1, and RPA70 proteins are essential components of the HR repair pathway [78]. RAD51 plays a role in the exchange between a damaged strand and its corresponding sister strand at sites of DSBs [79]. This protein is generally involved in various cellular processes, including mitosis, DNA replication, and DNA damage response [80], as well as in resumption of stalled replication forks [26]. Expectedly, the absence of *Rad51* in mouse MII oocytes resulted in premature separation of sister chromatids, aneuploidy, and chromosomal fragmentation, which may be attributed to defects in the HR process [81]. Its deficiency also resulted in impaired oocyte maturation in pigs [82].

The present study revealed the expression of RAD51 in oocytes and granulosa cells of follicles at all stages, from primordial to antral, as well as in other ovarian cells. This expression pattern indicates that RAD51 is involved in DSB repair, DNA replication, the other cellular processes in these cell types. The increasing expression of this protein in oocytes and granulosa cells of secondary, preantral and antral follicles, as well as in germinal epithelium and granulosa lutein cells in older ages, could contribute to repairing DSBs, which normally elevate during maternal aging.

In contrast to our finding, some studies have demonstrated a reduction in RAD51 expression in primordial oocytes of aged female rats and buffaloes [23, 83]. Similarly, the levels of *RAD51* mRNA decreased in GV-stage oocytes of old mice and humans in parallel with the accumulation of DSBs [28]. The mRNA and protein levels of RAD51 were found to reduce significantly in porcine oocytes undergoing in vitro aging (24 and 48 h) when compared to the controls [84]. However, an elevated *RAD51* mRNA level was observed in aged human cumulus cells [85]. This contradictory results for *RAD51* expression during maternal aging may be attributed to species-specific differences and using distinct techniques and materials employed for the analyses. Consequently, further studies are required to precisely determine the changes in RAD51 expression during ovarian aging and its functional status in aged ovarian cells, with a particular focus on oocytes and granulosa cells.

Another key component of HR repair, RPA70, specifically binds to ssDNA to form a heterotrimeric structure that effectively protects the extended 3’-end [86, 87]. RPA70 is additionally involved in various cellular processes, such as DNA replication and recombination, modulation of cell cycle progression and control of DNA damage checkpoints [31, 88]. In addition to its defined role in somatic cells, RPA proteins participate in facilitating meiotic recombination in female germ cells [89]. When *Rpa1* is mutated in mice, it leads to early embryonic lethality [90]. The present study revealed a significant decline in RPA70 levels in the follicles of both the early and late aged groups. This may be associated with the decreased DNA replication and transcription processes during aging [91], as RPA70 is known to be involved in these processes [92]. Further investigation is required to determine whether there is a potential association between decreased RPA70 expression in the follicles at late ages and female fertility loss during maternal aging.

It is established that the BRCA1 protein functions in conjunction with RAD51 in the repair of DSBs via HR pathway. Given that BRCA1 plays a pivotal role in DSB repair in primordial follicles [28], it can be postulated that functional loss of BRCA1 results in a reduction in the number of these follicles and an accumulation of DSBs during aging. However, no discernible alteration was observed in the DSB levels of granulosa cells in *Brca1*-null mice [28], indicating compensation of its absence by the functioning of similar proteins.

Zhang et al., (2015) observed a reduction in the number of BRCA1 foci in granulosa cells of primordial, primary, and secondary follicles in ovaries of aged rhesus monkeys [17]. Furthermore, BRCA1 foci number decreased in the nuclei of oocytes with age. There was age-related decrease in the *Brca1* mRNA level in the primordial follicles of rats [23]. The same group also revealed that *Brca1* mRNA level decreased in primordial follicles in adult buffaloes when compared to the young [24]. Similar to the former studies, we detected decreased BRCA1 levels in the follicles from primary to antral stages in older ages. Given the close association between BRCA1 and RAD51 proteins [93], it can be postulated that decreased BRCA1 expression may result from reduced RAD51 expression, at least in preantral and antral follicles.

The cNHEJ factors, KU70/KU80 heterodimer and XRCC4, are involved in the repair of DSBs that arise from irradiation (IR) and V(D)J recombination [94]. Oocyte-specific conditional knockout of the *Ku80* gene (herein referred to as *Xrcc5*) in mice caused no alterations in the number of healthy follicles, atretic follicles, or corpora lutea [95]. However, GV oocytes lacking *Ku80* were unable to repair DSBs, even at low levels, induced by etoposide [96], showing necessity of KU80 in repairing DSBs. Consequently, KU80, as a component of cNHEJ pathway, contributes to maintaining genomic integrity and proper oocyte maturation. The current study revealed a significant increase in KU80 levels with age, which is attributed to elevated expression in oocytes and granulosa cells of secondary, preantral, and antral follicles, as well as in stromal cells and granulosa lutein cells. In addition to its primary role in repairing increased DSBs in aged ovarian cells, KU80 may also play a role in preventing tumorigenesis [97] early senescence in these cells through its involvement in telomere maintenance [98].

The XRCC4 protein plays a role in maintaining genomic stability by participating in the repair of DSBs during oocyte maturation and early embryonic development [99]. The loss of XRCC4 function resulted in impaired DNA repair in oocytes, which subsequently led to the development of premature ovarian insufficiency (POI) [100]. It is worth noting that XRCC4 undergoes phosphorylation at its C-terminus by cyclin-dependent kinase 1 (CDK1) or polo-like kinase 1 (PLK1), which results in the inhibition of its DSB repair activity [101]. The absence of the *Xrcc4* gene in mice resulted in embryonic death, which was characterized by a high rate of apoptosis [102]. Its deficiency in mammalian cells impaired DSB repair and resulted in a high sensitivity to ionizing radiation [103]. In summary, these results indicate that maintaining XRCC4 at a stable level and non-phosphorylated state are essential for the promotion of DSB repair via the cNHEJ pathway. Otherwise, there would be a risk of the appearance of aging-related phenotypes, cellular demise, and increased apoptosis. In a study by Lin et al., (2021), it was observed that the mRNA levels of *XRCC4* were reduced in porcine oocytes that had undergone in vitro aging (24 and 48 h) in comparison to the controls. [84]. However, in the current investigation, we found an increase in XRCC4 levels in the growing follicles and stromal cells, granulosa lutein, and theca lutein cells of aged groups. This increase may contribute to rapidly repairing DSBs in the ovarian cells during aging. It remains elusive how in vitro and biological aging affect phosphorylation status of XRCC4 protein in ovarian cells.

Although this study reveals valuable insights, it is important to acknowledge that it is not without limitations. It would be beneficial to evaluate the remaining proteins that participate in the repair of DSBs by the HR and cNHEJ pathways in these ovaries at different postnatal ages. The measurement of cNHEJ and HR repair activity in the postnatal ovaries from early to aged periods, especially in follicles, could provide further insight into their potential efficacy in DSB repair. Also, the potential interactions among the HR and cNHEJ repair proteins can be investigated by performing double or more staining in ovarian cells during aging, with a particular focus on oocytes and granulosa cells. Finally, since we used our previous project’s paraffin blocks, there is no serum sample of the mice to measure oxidative levels and reproductive hormones such as estrogen and progesterone.

## Conclusion

In this study, we found that the β-GAL, cCASP3, γH2AX, RAD51, BRCA1, RPA70, KU80, and XRCC4 proteins exhibit follicle-specific expressional differences in the postnatal mouse ovaries from early to old ages (Supplementary Figs. 1–3). Given that these DSB repair proteins play a role in maintaining genomic integrity and cellular mechanisms in ovarian cells, including oocytes and granulosa cells, further studies are required to elucidate the molecular mechanisms underlying age-related changes in their expression. In particular, the potential effects of reproductive hormones (such as estrogen and progesterone), oxidative stress, loss of mitochondrial function, and telomer shortening should be examined. A comprehensive understanding of these molecular mechanisms may provide crucial insights into the understanding of reduced fertility in older women. Thus, novel strategies such as increasing or decreasing DSB repair-related gene expression may be discovered to prevent or delay the decline in female fertility in later reproductive life.

## Supplementary Information

Below is the link to the electronic supplementary material.

Supplementary file (DOCX 680 KB)

## Data Availability

The datasets generated during and/or analyzed during the current study are available from the corresponding author upon reasonable request.
