# Supplementary material for: The DNA double-strand break repair proteins γH2AX, RAD51, BRCA1, RPA70, KU80, and XRCC4 exhibit follicle-specific expression differences in the postnatal mouse ovaries from early to older ages
Source: J Assist Reprod Genet. 2024 Jul 18;41(9):2419–39. doi: 10.1007/s10815-024-03189-4 (PMC11405603; doi:10.1007/s10815-024-03189-4)
Supplement: Supplementary file 1 — Supplementary file (DOCX 680 KB) [file 10815_2024_3189_MOESM1_ESM.docx]

**Supplementary Figure Legends**

**
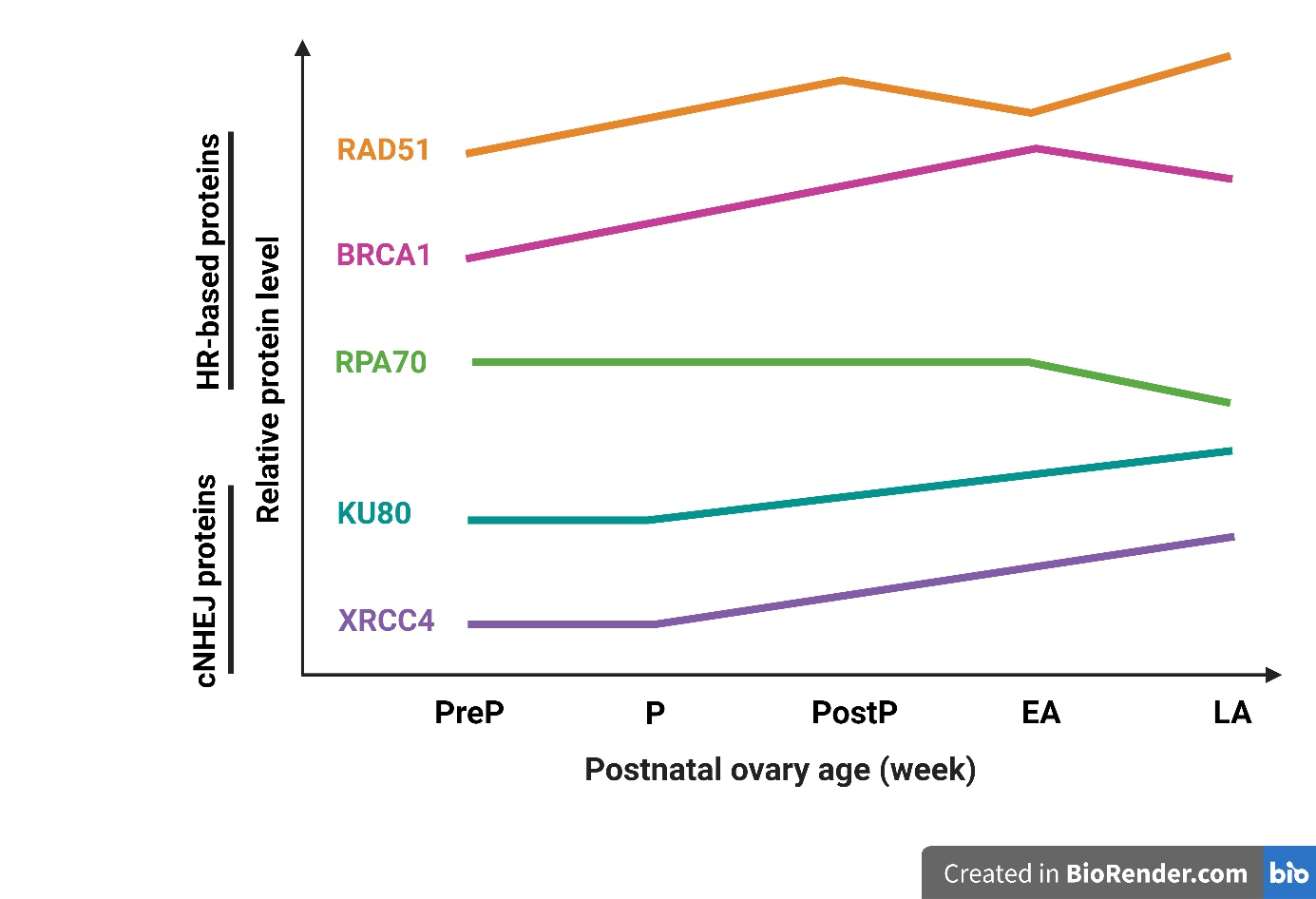
**

**Supplementary Figure 1.** The relative expression of the HR (RAD51, BRCA1, and RPA70) and cNHEJ (KU80 and XRCC4) proteins in the postnatal ovaries from the prepuberty to the late aged groups. PreP, Prepuberty; P, Puberty; PostP, Postpuberty; EA, Early aged; LA, Late aged. The line chart was created using the BioRender scientific illustration software (BioRender Company, Toronto, Canada).

**
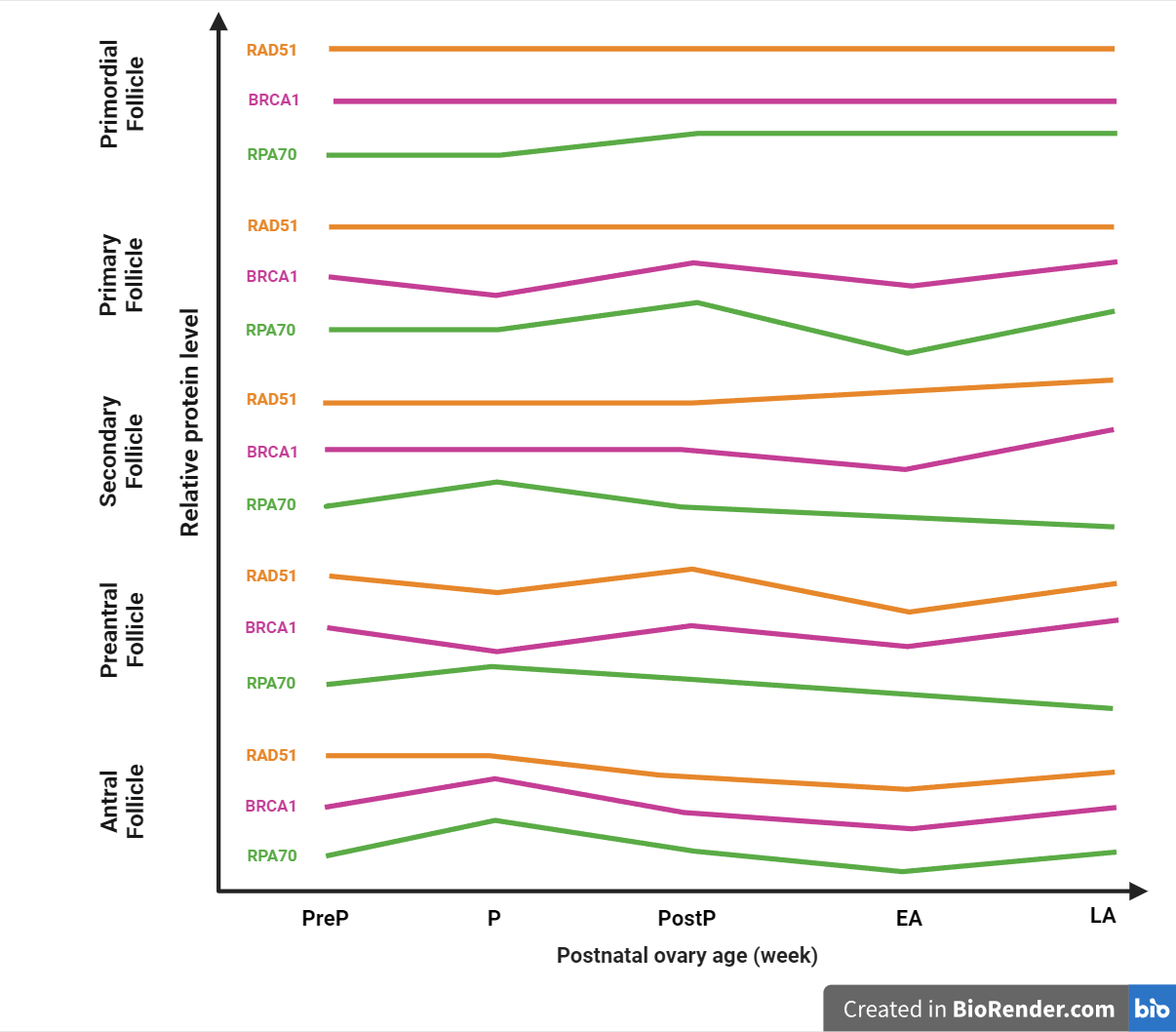
**

**Supplementary Figure 2.** The relative expression of the HR pathway-related proteins (RAD51, BRCA1, and RPA70) in the follicles from primordial to antral stages in the postnatal ovaries from the prepuberty to the late aged groups. PreP, Prepuberty; P, Puberty; PostP, Postpuberty; EA, Early aged; LA, Late aged. The line chart was created using the BioRender scientific illustration software (BioRender Company, Toronto, Canada).


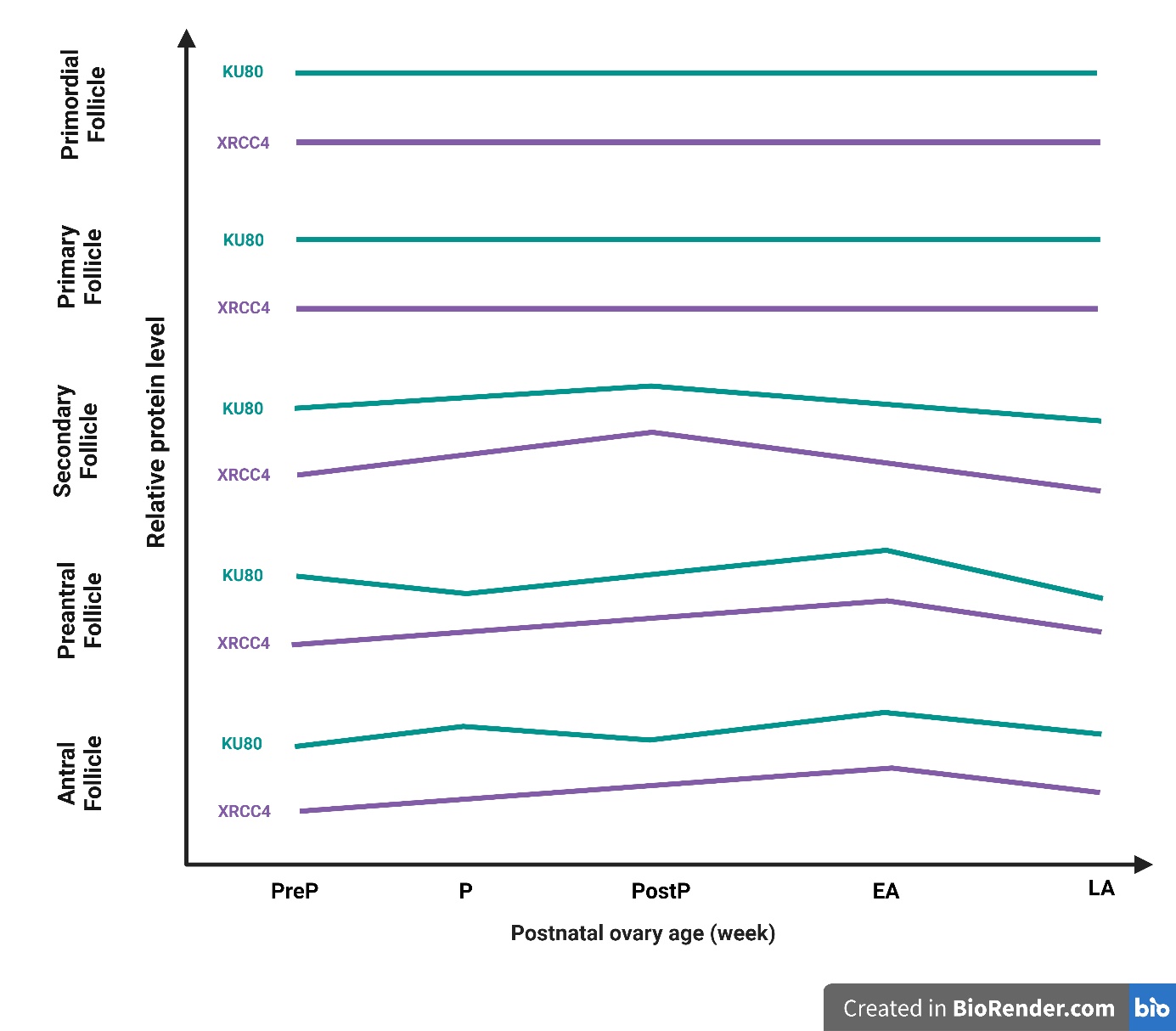


**Supplementary Figure 3.** The relative expression of the cNHEJ pathway-related proteins (KU80 and RPA70) in the follicles from primordial to antral stages in the postnatal ovaries from the prepuberty to the late aged groups. PreP, Prepuberty; P, Puberty; PostP, Postpuberty; EA, Early aged; LA, Late aged. The line chart was created using the BioRender scientific illustration software (BioRender Company, Toronto, Canada).
